# Generalized Tetanus in an Adult Patient

**DOI:** 10.7759/cureus.44958

**Published:** 2023-09-09

**Authors:** Karen Castaneda, Luis F Lemus, Ernesto Revelo

**Affiliations:** 1 Internal Medicine, San Rafael National Hospital, Santa Tecla, SLV; 2 School of Medicine, Dr. Jose Matias Delgado University, Santa Tecla, SLV; 3 General Medicine, Health Unit of Puerto de La Libertad, La Libertad, SLV

**Keywords:** resource limited country, wound complication, muscular spasms, generalized tetanus, tetanus vaccine, clostridium tetani

## Abstract

Tetanus is a vaccine-preventable disease that commonly occurs in under-resourced countries; clinically, it manifests as spontaneous muscle spasms and overall body rigidity, which can lead to autonomic dysfunction. The diagnosis of tetanus is primarily clinical, although laboratory testing is available, treatment of a clinical case should never be delayed. Management includes general support measures, prevention of complications, control of muscle spasms, and immunoglobulin. We present a patient from an underdeveloped region with a diagnosis of generalized tetanus after injury with a disc grinder. Clinical presentation of the patient, diagnostic studies performed, management, and outcome are discussed.

## Introduction

Tetanus is a vaccine-preventable disease caused by a bacteria called *Clostridium (C.) tetani*. It is a rare disease that commonly occurs in under-resourced countries [1]. *C. tetani* spores survive in the soil and cause infection by contaminated wounds. When the bacteria enters the body, it produces a toxin(tetanospasmin) that causes painful muscle contractions [2]. Reported cases of tetanus have declined by more than 95% worldwide; nowadays, sporadic cases occur in adults with an incomplete vaccination status [3]. Clinically, it manifests as spontaneous muscle spasms and overall body rigidity, which can lead to autonomic dysfunction and even death [4]. We present a patient from an underdeveloped region diagnosed with generalized tetanus after injury with a disc grinder. The clinical presentation of the patient, diagnostic studies performed, management, and outcome are discussed.

## Case presentation

A 63-year-old male from a rural background presented to the emergency department after suffering an injury with a disc grinder in his right knee. The injury at that time was 10 cm in length and 5 cm in width with irregular borders and active bleeding. The patient initially consulted with a primary care physician (PCP) at a rural health clinic who treated the injury with stitches and a course of antibiotics (amoxicillin 500 mg PO TID for 7 days). Following this, the patient presented to the ED of a second-level hospital one week after the initial injury with progressive right knee pain, hydrophobia, lockjaw, and sustained spasms of the face muscles that started two days ago. There was no past medical history, he denied taking medications daily, and there were no known allergies. The patient had incomplete immunization status. With regard to tetanus vaccination, his last dosage was unknown. Intramuscular tetanus vaccine was applied at a local health clinic one day before presenting to the ED.

**Figure 1:**
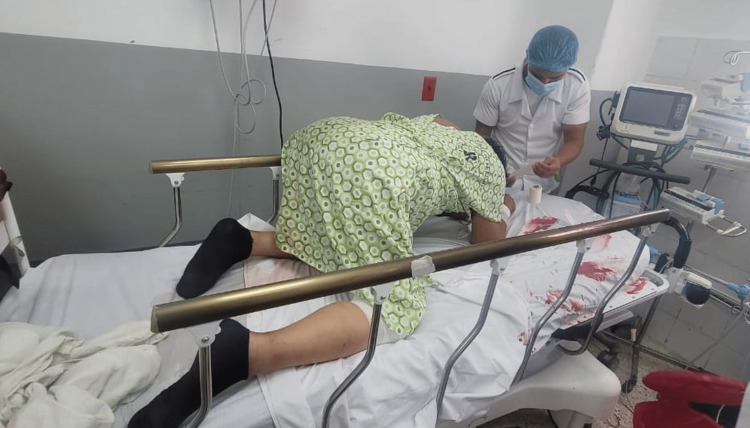
The patient was unable to maintain a supine position due to progressive back muscle spasms and stiffness of the neck.

On examination, his heart rate (HR) was 96 beats per minute, blood pressure (BP) was 110/75 mmHg, respiratory rate (RR) was 19 breaths per minute, oxygen saturation was 98% in room air, and he was afebrile with a temperature of 36.9 degrees Celsius. Physical examination showed trismus of the jaw, risus sardonicus, neck rigidity, increased tonicity of abdominal muscles, and a wound with stitches in the right knee, without dressings, with edema and drainage of seropurulent discharge. The right knee presented with redness and pain on palpation. Hypertonic extremities, generalized muscle spasms, and back arching spasms were seen (Figure 1). On neurological examination, the patient was oriented to time and place. Cranial nerves, deep tendon reflexes, strength, and sensitivity were normal.

**Figure 2:**
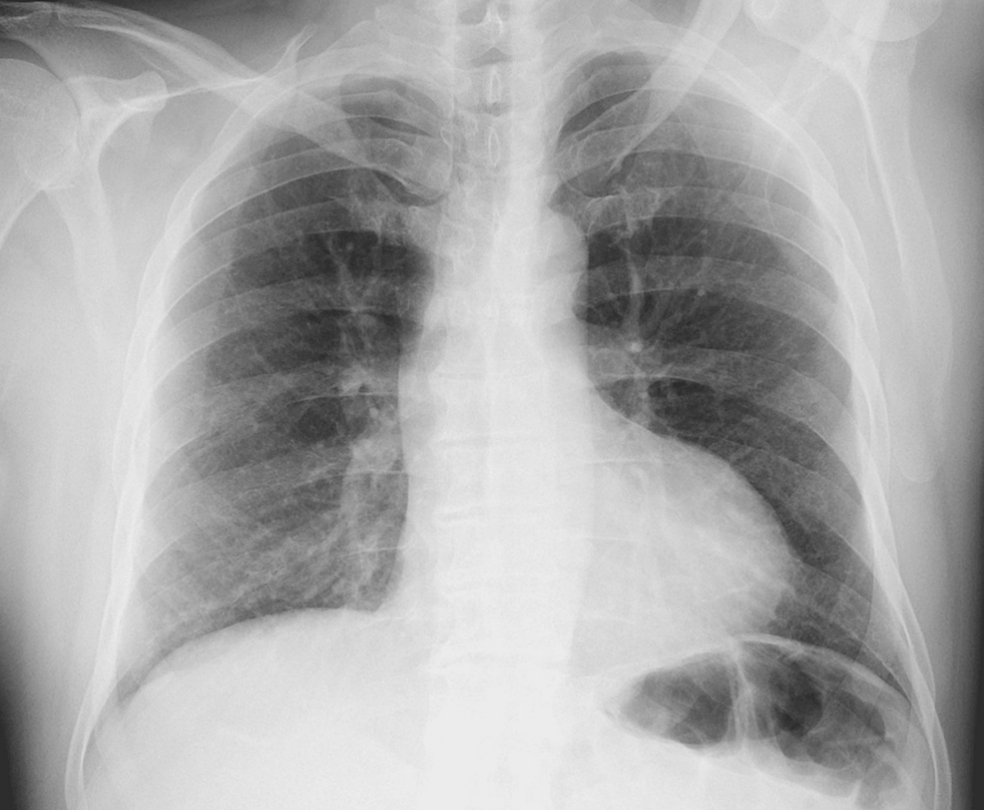
PA X-ray of the chest showed no consolidations and no infiltrations; a cardiac silhouette is observed with a cardiothoracic index of 0.49, and costophrenic and cardiophrenic angles visible. PA: posteroanterior

**Figure 3:**
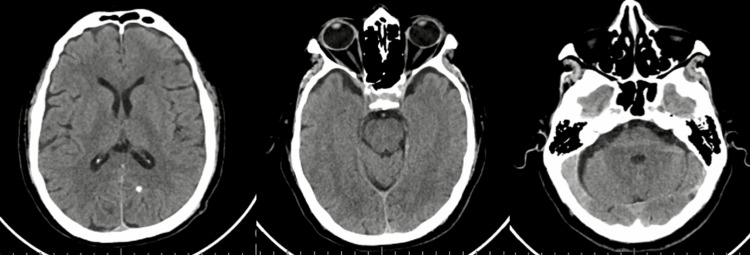
Axial views of the head CT scan were normal

The patient was admitted to the acute medical unit for further testing, with a differential diagnosis of meningoencephalitis post-vaccine and septic arthritis of the right knee. Tetanus was not considered due to the time from vaccine application to the onset of symptoms. Chest X-ray (Figure 2), CT scan of the brain (Figure 3), and lumbar puncture were done. His initial laboratory results are shown in Tables 1-2, blood, urine, and CSF cultures were also sent.

**Table 1:** Complete blood count MCV: mean corpuscular volume, WBCs: white blood cells

Test	Result	Reference range
Hemoglobin (g/L)	13.6	12-16
Hematocrit (%)	39.6	36-48
MCV (fL)	87.8	80-100
WBCs (x10^3^/uL)	11,530	5,000-10,000
Neutrophils count (%)	90	55-65
Platelets (×10^3^/uL)	453	150,00-400,000

**Table 2:** Renal profile and electrolytes BUN: blood urea nitrogen

Test	Result	Reference range
Sodium (mEq/L)	142	135-145
Potassium (mEq/L)	4.5	3.5-5.5
Chloride (mEq/L)	109	101-111
BUN (mEq/L)	21	7-21
Creatinine (mg/dL)	1.2	0.6-1.3
Glucose (mg/dL)	159	70-105
C-reactive protein	10.1	0-0.5

**Figure 4:**
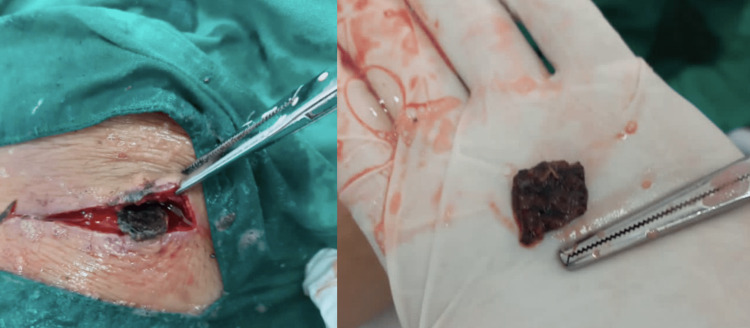
A foreign body in the right knee, measuring about 2 x 1 cm in diameter was extracted

**Figure 5:**
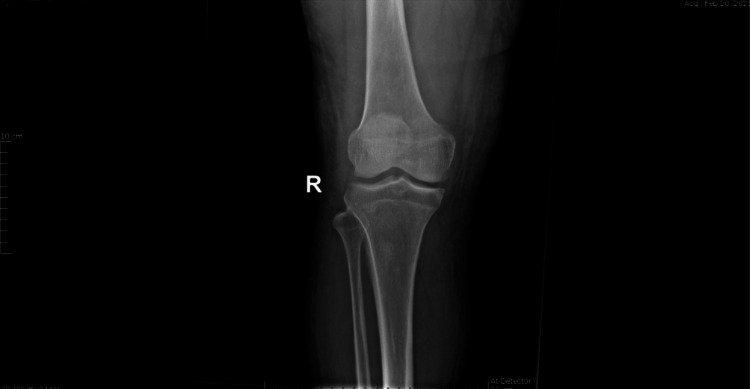
AP X-ray of the right knee showed no osteolytic changes PA: posteroanterior

General surgery was consulted, and they managed with the removal of stitches, where bloody purulent fluid was observed and a culture sample was negative. The presence of three fragments of foreign body, the largest estimated as 2 x 1 cm was extracted( Figure 4). A right knee X-ray was done (Figure 5).

Blood cultures taken on the date of admission came back positive for *Clostridium tetani* while other cultures were negative. After further evaluation by infectious disease the diagnosis of generalized tetanus was done and the patient was started on penicillin G 4 million units every four hours, metronidazole 500 mg IV QID, and intramuscular tetanus immunoglobulin. On the second day of hospitalization, the patient began to deteriorate clinically and the decision to begin mechanical ventilation was made for airway protection. After 12 days of stay in the ICU, the patient developed septic shock, acute kidney injury (AKI) stage 3, and abdominal obstruction. The patient was pronounced dead secondary to multiorgan system failure after 15 days in the ICU.

## Discussion

In the United States, nearly all cases of tetanus occur among people who have never received a tetanus vaccine or didn’t stay up to date on their booster shots every 10 years [5]. From 2000-2019, more than 55% of the 579 reported cases were among people 20 through 59 years of age [5]. According to the World Health Organization, data published 202 deaths caused by tetanus in El Salvador reached 11, with a mortality rate by age of 0.13 per 100,000 populations [6].

Tetanus is a neuromuscular disease in which tetanospasmin, an exotoxin released by *Clostridium tetani* causes muscle spasms. This bacteria can be found globally, usually recovered from soil, more commonly in third-world countries [7]. The incubation period of tetanus varies between three and 21 days after infection, with most cases occurring within 14 days [8]. Tetanus arises from minor skin cuts or abrasion in the majority of cases and in 20-50% with no obvious entry site [1]. In our case, the patient had a clear point of entry after being in contact with a disc grinder and a retained foreign body in his right knee. Presenting features on admission to the hospital for tetanus are trismus (93-98%), generalized muscle tension (94-95%), muscle stiffness (96%), dysphagia( 83%), dyspnoea (7%), muscle spasms (46-80%), body temperature > 38.4 degrees Celsius, and pulse of >120 beats per minute(34%) [1].

Key clinical features of generalized tetanus include at least two of the following for the diagnosis: trismus (painful contractions of the neck muscles leading to facial spams), painful muscular contraction of the trunk muscles, and generalized spasms. Diagnosis of tetanus is primarily a clinical diagnosis [9]. The differential diagnosis for generalized muscle spams includes strychnine poisoning and dystonic reactions to medications such as phenothiazines and metoclopramide [1]. Laboratory testing is also available to support the diagnosis but treatment of a clinical case should never be delayed. Laboratory testing includes wound samples, isolates from culture, and serum antibody testing [9].

Most cases of tetanus occur in resource-limited settings and evidence to guide optimal management is scarce, and it highly depends on the institution [1]. Management overall includes the prevention of toxin uptake, control of muscle spasms, and supportive care. Wounds require cleaning and debridement; antitoxin and antibiotics are the only specific therapies available [1]. Post-exposure prophylaxis following an injury can prevent clinical tetanus and timely administration is necessary [10]. Prophylaxis depends on the nature of the wound and vaccination history; immunosuppressed patients should always be managed as they have incomplete vaccination [11]. If left untreated, clinical tetanus usually presents between four and 21 days after inoculation [11]. Despite adequate management in the ICU with the best available resources our patient succumbed to the disease.

## Conclusions

Despite the fact that tetanus occurs extremely rarely, cases are still reported in under-resourced countries, particularly in patients who are not up to date with their vaccinations, as we observed in this patient. Therefore, physicians in countries where there are still reported cases should know about the presentation of symptoms, diagnostic process, progression of the disease, and management of clinical tetanus. It is important to remind healthcare professionals about tetanus prophylaxis in routine wound management, as delay in prophylaxis can result in clinical tetanus. This case highlights the importance of the early diagnosis of this disease owing to its high mortality.

## References

[1] Yen LM, Thwaites CL (2019). Tetanus. Lancet.

[2] Hassel B (2013). Tetanus: pathophysiology, treatment, and the possibility of using botulinum toxin against tetanus-induced rigidity and spasms. Toxins (Basel).

[3] (2023). Tetanus surveillance. CDC. https://www.cdc.gov/tetanus/surveillance.html.

[4] Almas T, Niaz MA, Zaidi SM (2021). The spectrum of clinical characteristics and complications of tetanus: a retrospective cross-sectional study from a developing nation. Cureus.

[5] (2023). Tetanus: for clinicians. CDC. https://www.cdc.gov/tetanus/clinicians.html.

[6] Tétanos en El Salvador (2023). Tétanos en El Salvador. World Life Expectancy. https://www.worldlifeexpectancy.com/es/el-salvador-tetanus.

[7] Hallit RR, Afridi M, Sison R, Salem E, Boghossian J, Slim J (2013). Clostridium tetani bacteraemia. J Med Microbiol.

[8] (2023). Tetanus. WHO. https://www.who.int/news-room/fact-sheets/detail/tetanus.

[9] (2023). Tetanus: advice for health professionals. GOV.UK. https://www.gov.uk/government/publications/tetanus-advice-for-health-professionals.

[10] Miyagi K, Shah AK (2011). Tetanus prophylaxis in the management of patients with acute wounds. J Plast Reconstr Aesthet Surg.

[11] Collins S, White J, Ramsay M, Amirthalingam G (2015). The importance of tetanus risk assessment during wound management. IDCases.

